# Persistent Detection and Infectious Potential of SARS-CoV-2 Virus in Clinical Specimens from COVID-19 Patients

**DOI:** 10.3390/v12121384

**Published:** 2020-12-03

**Authors:** Michael Zapor

**Affiliations:** Veterans Affairs Medical Center, Martinsburg, WV 25405, USA; michael.zapor@va.gov; Tel.: +1-304-263-0811 (ext. 3490)

**Keywords:** coronavirus, SARS-CoV-2, COVID-19, shedding

## Abstract

The Severe Acute Respiratory Syndrome Coronavirus (SARS-CoV-2) that emerged in December 2019 as the causative agent of Coronavirus 2019 (COVID-19) and was declared a pandemic by the World Health Organization in March 2020 has several distinctive features, including extensive multiorgan involvement with a robust systemic inflammatory response, significant associated morbidity and mortality, and prolonged persistence of viral RNA in the clinical specimens of infected individuals as detected by Reverse Transcription Polymerase Chain Reaction (RT-PCR) amplification. This review begins with an overview of SARS-CoV-2 morphology and replication and summarizes what is known to date about the detection of the virus in nasal, oropharyngeal, and fecal specimens of patients who have recovered from COVID-19, with a focus on the factors thought to contribute to prolonged detection. This review also provides a discussion on the infective potential of this material from asymptomatic, pre-symptomatic, and convalescing individuals, to include a discussion of the relative persistence and infectious potential of virus in clinical specimens recovered from pediatric COVID-19 patients.

## 1. Introduction

Named for the crown-like arrangement of glycoproteins on their capsid, the coronaviruses comprise a family within the order Nidovirales and consist of four genera: Alphacoronavirus, Betacoronavirus, Gammacoronavirus, and Deltacoronavirus. Coronaviruses are common in birds and mammals (with the greatest diversity in bats), and human infections are caused by two alpha- (i.e., HCoV-229E and HCoV-NL63) and several beta- (e.g., HCoV-OC43 and HCoV-HKU1) species [1]. Severe Acute Respiratory Syndrome coronavirus (SARS-CoV) and Middle East Respiratory Syndrome coronavirus (MERS-CoV) are also beta-coronaviruses. Coronaviruses are ubiquitous and along with rhinoviruses, parainfluenza, metapneumovirus, and respiratory syncytial virus, cause most community-acquired upper respiratory tract infections (i.e., the common cold) [2]. As with other respiratory viruses, coronaviruses occasionally cause more severe illness, with individuals at the extremes of age (i.e., infants and the elderly), as well as those with comorbid pulmonary disease (e.g., chronic obstructive pulmonary disease), or immune compromising conditions (e.g., hematopoietic stem cell transplant or HIV infection) at increased risk. Certain coronavirus species (e.g., HCoV-OC43, SARS-CoV, and MERS-CoV) also are associated with more severe infection [3]. Except for SARS-CoV and MERS-CoV, there has not been much interest in producing coronavirus vaccines. This derives from the fact that most coronaviruses: (1) cause mild, self-limiting illness; (2) are difficult to replicate in tissue culture; (3) display antigenic variation; and also (4) vaccine trials with at least one animal coronavirus demonstrated a worse outcome upon challenge with the virus (a problem similarly posed by dengue virus) [4]. Although some medicines, including antivirals and chloroquine, have demonstrated potent in vitro antiviral activity against tested coronaviruses (i.e., SARS-CoV, HCoV-229E, and HCoV-OC43), randomized controlled clinical trials have not demonstrated efficacy and treatment is supportive. As with other respiratory viruses (such as rhinoviruses), coronaviruses are transmitted by respiratory aerosol, and the mainstay of prevention is handwashing, respiratory hygiene, and disinfection of fomites.

In December 2019, a novel coronavirus, provisionally designated 2019-NCoV (i.e., 2019 Novel Coronavirus), emerged from Wuhan, a city in the Hubei Province of China [5]. In the ensuing two months, the virus spread rapidly throughout China, causing respiratory illness of varying severity. The incubation period was four days, and among those hospitalized, the most common symptoms were fever (88.7%) and a cough (67.8%). Ground glass opacifications on chest computed tomography (CT) (56.4%) and lymphocytopenia (83.2%) were also common features [6]. Owing to its similarities with the SARS coronavirus that emerged from China in 2003 (SARS-CoV), to include 76.47% amino acid sequence homology in its spike (S) protein [7], as well as its similar recognition and binding to the angiotensin converting enzyme-2 (ACE-2) receptor [8], 2019-NCoV was redesignated SARS-CoV-2 by the International Committee for the classification of viruses [9]. On January 13 2020, the first lab-confirmed SARS-CoV-2 coronavirus disease (COVID-19) case outside of China was announced by the World Health Organization (WHO) in a patient who had traveled to Thailand from Wuhan [10], and on 16 January a second imported case, also in a traveler from Wuhan, was reported by the Japanese Ministry of Health, Labor and Welfare, prompting the Pan American Health Organization/WHO Regional office for the Americas (PAHO/AMRO) to issue its first epidemiological alert on the novel coronavirus [11]. By the end of the month, cases had also been reported in Europe, the United States, and Southeast Asia, and the WHO had declared the outbreak a public health emergency of international concern [12]; and in a Tweet on 11 March 2020, the WHO announced that the outbreak could “be characterized as a pandemic” [13]. 

By 28 June 2020, ten million people were reported to have been infected globally, with twenty million cases reported by 10 August, thirty million cases by 17 September, and forty million cases by 19 October 2020 [14]; and as of 23 November 2020, there have been 59,127,000 COVID-19 cases documented with 1,396,017 deaths in 215 countries, territories, and conveyances [15]. Although much about the virus and the management of infected patients remains to be learned, it is apparent that there are distinct clinical features that are highly conserved among sicker COVID-19 patients. Included among these are hypoxia and dyspnea with rapid progression to acute respiratory distress syndrome (ARDS), hypercoagulability and coagulopathy, cardiovascular complications including myocardial infarction and arrhythmias, acute renal failure sometimes requiring hemodialysis, and delirium [16,17,18,19,20]. Additionally, radiographic and laboratory abnormalities are highly conserved among COVID-19 patients. The former typically consists of rapidly progressive ground glass opacifications [21]. Among the latter, an elevated D-dimer as well as elevated inflammatory reactants including ferritin, procalcitonin, erythrocyte sedimentation rate, and C-reactive protein are common [22]. Moreover, the persistence of viral RNA in clinical samples, as detected by reverse transcription polymerase chain reaction (RT-PCR) amplification, is well-documented among COVID-19 patients [23]. This phenomenon raises many questions about both the clinical management of recovered patients in whom viral RNA is still detectable, as well as the public health implications of a persistently positive RT-PCR assay. 

Following is an overview of SARS-CoV-2 morphology and replication and a summary of what is known to date about the detection of the virus in nasal, oropharyngeal, and fecal specimens of patients who have recovered from COVID-19, with a focus on the factors thought to contribute to prolonged detection. This review also provides a discussion on the infective potential of this material from asymptomatic, pre-symptomatic, and convalescing individuals, to include a discussion of the relative persistence and infectious potential of the virus in clinical specimens recovered from pediatric COVID-19 patients. It should be noted that the term “clinical shedding” is used throughout this review to refer to the detection of viral RNA by reverse transcription polymerase chain reaction amplification (i.e., RT-PCR positivity) in clinical specimens and its use is intended to avoid confusion with cellular shedding (i.e., the release of virions from infected cells). 

## 2. SARS-CoV-2 Entry and Replication

SARS-CoV-2 is a positive-sense single-stranded RNA virus. Its genome is ~30 kb which, like those of other coronaviruses, consists of genes for four structural proteins including surface (S), envelope (E), membrane (M), and nucleocapsid (N) proteins, as well as six accessory proteins, encoded by genes on open reading frames ORF3a, ORF6, ORF7a, ORF7b, and ORF8. Additional open reading frames encode a host of nonstructural proteins, including those that facilitate replication and transcription and others that enable the virus to evade the host immune response [24]. Like all viruses, SARS-CoV-2 relies on the replicative machinery of a vulnerable host cell to make copies of its genome, a process that begins with cell binding and entry. Attachment to and entry of SARS-CoV-2 into susceptible cells is mediated by the spike protein, which consists of two subunits: S1 and S2 [25]. The S1 subunit binds to angiotensin converting enzyme, ACE-2, a receptor on the host cell that is distinct from ACE-1 (the enzyme targeted by ACE inhibitors such as lisinopril, enalapril, and ramipril). The ACE-2 protein is widely distributed throughout the human body, and most abundantly expressed in the lung type II alveolar cells, enterocytes of the gastrointestinal tract, endothelial cells, smooth muscle cells, cortical neurons, and glial cells [26]. As S1 subunits bind to the membrane-bound ACE-2 protein molecules, the virus becomes enveloped in an endosome. Cell entry then continues by either of two processes. In the first, transmembrane protease, serine 2 (TMPRSS2) cleaves the S1 subunits from the S2 subunits and cleaves the ACE-2 proteins. The endosome is then endocytosed, and the virus is subsequently released into the cytoplasm after acidification or through the proteolytic action of cathepsins. Alternatively, TMPRSS2 effects an irreversible conformational change of the S2 subunits, and the virus fuses to the cell membrane. 

**Figure 1:**
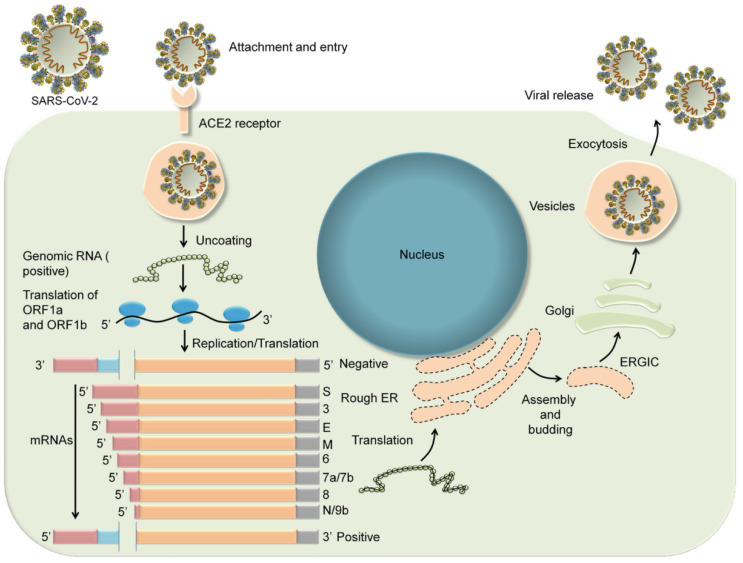
Entry and replication of SARS-CoV-2 in host cells. Reproduced with kind permission from Shailendra Saxena of King George’s Medical University (KGMU), Lucknow, India [27].

Entry of the virus into the host cell and release from the endosome is followed by uncoating of the virus and release of viral RNA into the cytoplasm where it undergoes translation. The translation products include the replicase polyproteins pp1a and pp1ab that undergo further cleavage into smaller proteins including RNA-dependent RNA polymerase, helicase, and nonstructural proteins nsp3, nsp4, and nsp6. During translation, ribosomal frame shifting generates genomic and sub genomic moieties by discontinuous transcription. The coronavirus replication–transcription complex is then anchored to the intracellular membrane of the endoplasmic reticulum by nsp3, nsp4, and nsp6 to form double membrane vesicles. RNA-dependent RNA polymerase and helicase then drive the synthesis of sub genomic RNA from which structural and accessory proteins are produced, including the S, M, and E proteins, which are inserted into the endoplasmic reticulum and then transported to the endoplasmic reticulum-Golgi intermediate compartment (ERGIC). In contrast, the N protein binds the viral genomic RNA in the cytoplasm to form the nucleocapsid. The virions are then assembled in the ERGIC and released in vesicles from the cell by exocytosis (Figure 1) [27]. 

## 3. Duration of SARS-CoV-2 RT-PCR Positivity and Factors Associated with Prolonged Clinical Shedding

Prolonged clinical shedding has been described for several respiratory viruses, including SARS-CoV [28] and MERS-CoV [29], and the persistence of SARS-CoV-2 in respiratory secretions, as detected by RT-PCR amplification, was described early in the pandemic [30,31]. One of the earliest studies of infected patients reported the median and absolute duration of RT-PCR positivity to be 20 days and 37 days, respectively [32]. However, more recent reports describe the persistence of the virus for more than 60 days with a median duration of RT-PCR positivity of more than 30 days [33]. There are several factors that appear to correlate with prolonged clinical shedding, including severe illness. In one study, the median duration of the virus in respiratory specimens from patients with severe disease (21 days, 14–30 days) was significantly longer than in patients with mild disease (14 days, 10–21 days; *p* = 0.04) [34]. These findings are consistent with reports of longer clinical shedding times in ICU patients than in patients not in an ICU [35]. Moreover, the mean viral load in patients with severe illness appears to be significantly higher (around 60 times higher in one study) than that of patients with mild illness, suggesting that higher viral loads might be associated with a more severe clinical course [36]. However, at least one other study found no significant correlation between severity of illness and viral load [37]. Additionally, one study found an inverse correlation between disease severity and duration of RT-PCR positivity [38]. 

**Figure 2:**
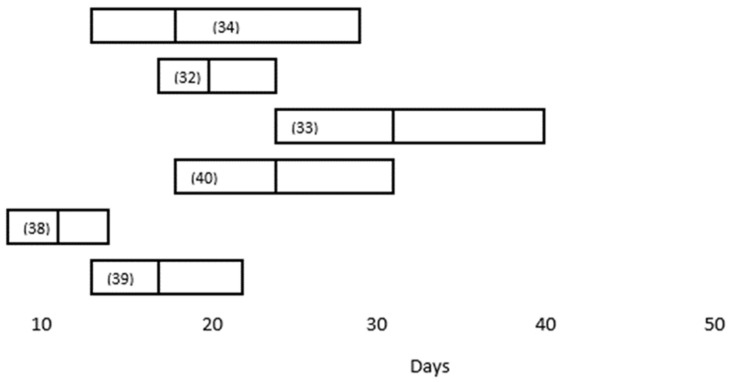
Duration of SARS-CoV-2 RT-PCR positivity in respiratory specimens reported in studies of adult COVID-19 patients cited in this review. Vertical lines represent the median duration, and the boxes represent the interquartile range. The study reference numbers are in parentheses. See text for additional details.

Several research groups have looked at the factors associated with viral persistence in COVID-19 patients (Table 1) [32,33,34,35,39,40,41]. In a retrospective study of 101 COVID-19 patients consecutively hospitalized in Beijing’s YouAn Hospital, the median duration of RT-PCR positivity was 11 days (8–14.3 days); and the factors associated with prolonged clinical shedding (defined as >11 days) included fever (temperature > 38.5 °C) (OR 5.1, 95% CI: 1.5–18.1), corticosteroid use (OR 6.3, 95% CI: 1.5–27.8), and time from onset to hospitalization (OR 1.8, 95% CI: 1.19–2.7) [39]. In this study, severe disease (defined as any of the following: respiratory distress, respiratory rate ≥ 30 beats/min; resting oxygen saturation ≤93%; arterial blood oxygen partial pressure/oxygen concentration ≤ 300 mmHg [1 mmHg = 0.133 kPa]; progression of infiltrates on chest imaging by more than 50% within 24–48 h) was initially associated with prolonged clinical shedding. However, the association with disease severity was not evident after multivariate regression analysis. In a similar retrospective study of 113 hospitalized patients at two other hospitals in China, the median duration of SARS-CoV-2 RNA detection from illness onset was 17 days (IQR, 13–22 days). Prolonged clinical shedding (defined as >15 days) was seen in 76 patients (67.3%) and was associated with the male gender (OR, 3.24; 95% CI, 1.31–8.02), invasive mechanical ventilation (OR, 9.88; 95% CI, 1.11–88.02), and time from illness onset to hospital admission (odds ratio [OR], 1.30; 95% confidence interval [CI], 1.10–1.54; *p* = 0.002) [41]. As with several other studies, after multivariate analysis disease severity was not an independent risk factor for viral persistence. A comparison of the median duration of SARS-CoV-2 RT-PCR positivity in respiratory specimens reported in these studies is depicted in Figure 2. 

## 4. Persistent Detection of SARS-CoV-2 in Feces

Although SARS-CoV-2 is primarily transmitted via respiratory secretions and COVID-19 generally manifests as pneumonia, the widespread distribution of ACE-2 receptors makes COVID-19 a systemic infection. Enterocytes of the small bowel abundantly express the receptor on their brush borders and may become infected by the movement of virions from the airways into the gastrointestinal tract (e.g., by expectoration or mucociliary clearance) [42]. In a mechanism elucidated by Wrapp et al. [43], human proteases such as TMPRSS2 (transmembrane protease serine 2) and furin then cleave the polybasic bonds between the S1 and S2 subunits of the spike protein, resulting in a separation of the two into a pincer-like configuration. The S1 subunit then binds the peptidase domain of ACE-2 and the S-2 subunit effects fusion with the cell membrane, which is then followed by viral endocytosis [43]. This is likely clinically relevant, because necropsied mice infected with SARS-CoV-2 demonstrate enterocyte desquamation, edema, small vessel dilation, lymphocyte infiltration, as well as mesenteric lymph node hemorrhage and necrosis [42]. Moreover, COVID-19 patients may have gastrointestinal complaints including diarrhea, either preceding, along with pneumonia [44], or as a sole clinical manifestation of SARS-CoV-2 infection [45]. Whether this is due to direct cytopathic effects, a systemic inflammatory response, or some other mechanism (e.g., disruption of trans-membrane transporters or of the gut microbiome) has yet to be elucidated. 

In addition to the clinical implications, gut involvement by SARS-CoV-2 raises the role of fecal shedding in transmitting the virus. In one systematic review of 55 studies (1348 patients), nearly half of collected stool samples had detectable virus. Moreover, the duration of fecal RT-PCR positivity (median 19 days) was significantly longer (*p* < 0.001) than that of respiratory RT-PCR positivity (median 14 days) [46]. In another meta-analysis, more than half of fecal samples had detectable virus for up to 70 days after the onset of symptoms and as long as 33 days (mean 12.5 days) after the virus was no longer detected in respiratory samples [47]. Despite the persistence of virus in the feces of infected individuals as detected by RT-PCR amplification, the infectious potential of fecal samples is uncertain. At least one study found quantitative titers to be below those of nasopharyngeal fluids and generally lower than those of enteric viruses such as norovirus and adenovirus [48]. Nonetheless, the presence of virus in the feces of COVID-19 patients raises concern for fecal–oral transmission, especially in situations in which adequate sanitation infrastructure is lacking, and the presence of SARS-CoV-2 in wastewater has been reported [49,50]. In this regard, young children may represent an important demographic, given their proclivity for unhygienic practices such as not washing their hands after stooling, sucking on their fingers, etc., and prolonged fecal RT-PCR positivity by pediatric COVID-19 patients has been described [51,52,53].

## 5. Persistent Detection of SARS-CoV-2 in Other Bodily Fluids

Extra-pulmonary tissue tropism has been documented for several coronaviruses including severe acute respiratory syndrome virus (SARS-CoV-1) and Middle East respiratory syndrome corona virus (MERS-CoV) [54,55] and it is increasingly apparent that SARS-CoV-2 is also organotropic, causing multi-system illness. Therefore, it is not surprising that acute kidney injury has been reported in more than a quarter of critically ill COVID-19 patients [56,57]. Although this may derive in part from hemodynamic instability and cytokine storm, several studies suggest a role for viral-mediated renal cytotoxicity; and in postmortem studies of COVID-19 patients, coronaviruses were identified in all kidney compartments examined, with apparent preferential targeting of glomerular cells [58]. In one meta-analysis of thirty studies in which the urine of COVID-19 patients was tested for virus using RT-PCR, the incidence of viruria was 8%, compared to 21.3% and 39.5% for blood and stool, respectively [59]. Although the infectious potential of SARS-CoV-2 in urine has yet to be elucidated, viable virus has been recovered from the urine of some COVID-19 patients, suggesting a possible role for genitourinary transmission [60], and at least one study has looked at the aerosolization of the virus from urinal flushing [61]. To date, however, viral RNA but not viable virus has been recovered from seminal fluid, and sexual transmission of SARS-CoV-2 has not been documented [62]. Similarly, viral RNA has been detected in tears, vomitus, and bile fluid, but the clinical significance of this has yet to be determined [63,64,65]. 

## 6. Infectious Potential of Clinically Shed SARS-CoV-2

The presence of SARS-CoV-2 RNA in respiratory secretions and other bodily fluids, as detected by RT-PCR, does not necessarily indicate viable virus; and their infectious potential is an area of active research with important public health implications. However, it is increasingly evident that the risk to others posed by post-convalescent COVID-19 patients may be negligible. For example, in one study of healthcare workers self-isolating due to persistent RT-PCR positivity up to 55 days after the onset of symptoms, no viable virus was recoverable in 29 of 29 nasopharyngeal/oropharyngeal samples tested [66]. In another similar study of 48 patients who had detectable viral RNA more than two weeks out from symptom onset, no virus could be recovered from any nasopharyngeal or salivary swab cultures [67]. The precise duration of infectivity likely varies, and the impact of several factors (such as viral load) is being studied. Nonetheless, data from a number of studies suggest that the risk posed by respiratory secretions is significantly reduced ten days after symptom onset [68], although viable virus may persist for as much as 15 days in saliva, urine and stool [69]. 

**Figure 3:**
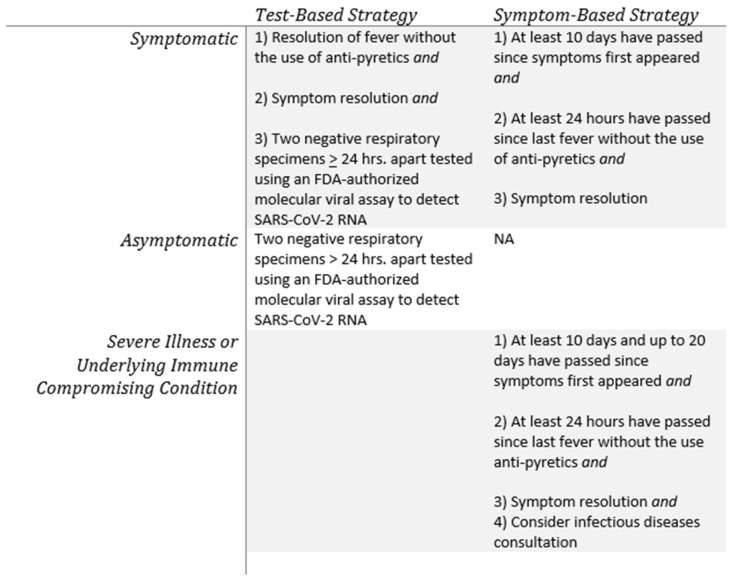
Summary of the Centers for Disease Control and Prevention (CDC) guidelines for the discontinuation of transmission-based precautions and disposition of patients with COVID-19 in healthcare settings. Recognizing that recovered COVID-19 patients may persistently shed inert non-infectious virus into clinical specimens, the CDC generally recommends a symptom-based rather than a test-based approach for symptomatic individuals [70]. (NA: Not applicable).

Based on these and similar studies, the Centers for Disease Control and Prevention (CDC) changed its guidance in August 2020 regarding the discontinuation of transmission-based precautions and disposition of patients with COVID-19 in healthcare settings from a test-based strategy to a symptom-based approach (Figure 3). According to the updated guidelines, transmission-based precautions for patients who are mildly to moderately ill and who are not severely immunocompromised may be discontinued when: (1) at least ten days have passed since symptoms first appeared; (2) at least twenty-four hours have passed since the last fever without the use of anti-pyretics; and (3) symptoms have improved. For patients with severe or critical illness or who are severely immunocompromised, the revised CDC guidelines recommend waiting at least ten and up to twenty days before discontinuing precautions. These guidelines acknowledge that prolonged clinical shedding of nonculturable virus occurs, and they replace previous guidelines that relied on negative RT-PCR results to clear a patient from transmission-based precautions [70].

## 7. Infectivity of SARS-CoV-2 Shed by Pre-Symptomatic and Asymptomatic Infected Individuals

In a June 8 press briefing, the World Health Organization’s (WHO) coronavirus technical lead stated that asymptomatic transmission of the SARS-CoV-2 virus was “very rare.” The comment, which seemingly suggested that infected people without symptoms were not spreading the disease, generated confusion about the role of wearing masks, social distancing, and sheltering in place—something the public has been exhorted to do in order to curtail asymptomatic spread of the virus. This was followed by a clarifying comment from the WHO the next day that the use of the term “very rare” had been a “miscommunication” and had been based on a small number of studies done in “member states” that followed the contacts of infected but asymptomatic individuals [71].

A role for asymptomatic clinical shedding in new COVID-19 cases is in fact suggested by several studies. In one study of 94 infected patients and another 77 documented cases of transmission, an estimated 44% of transmission occurred during the pre-symptomatic period, with infectiousness starting from 2.3 days (95% CI, 0.8–3.0 days) before symptom onset and peaking at 0.7 days (95% CI, −0.2–2.0 days) before symptom onset [72]. In another study published in the New England Journal of Medicine that documented transmission of SARS-CoV-2 in a skilled nursing facility in King County, Washington, 56% of residents with positive test results were asymptomatic at the time of testing and “most likely contributed to transmission” [73]. In an accompanying editorial, the authors claim that the study shows that “asymptomatic persons are playing a major role in the transmission of SARS-CoV-2” [74]. Lastly, in a comprehensive meta-analysis, researchers at the Scripps Research Translational Institute reviewed the data from sixteen international studies in which a total of 45,394 individuals were screened for SARS-CoV-2 and found that of the 6738 individuals who tested positive, 40–45% remained asymptomatic [75].

## 8. SARS-CoV-2 Clinical Shedding by Children

**Figure 4:**
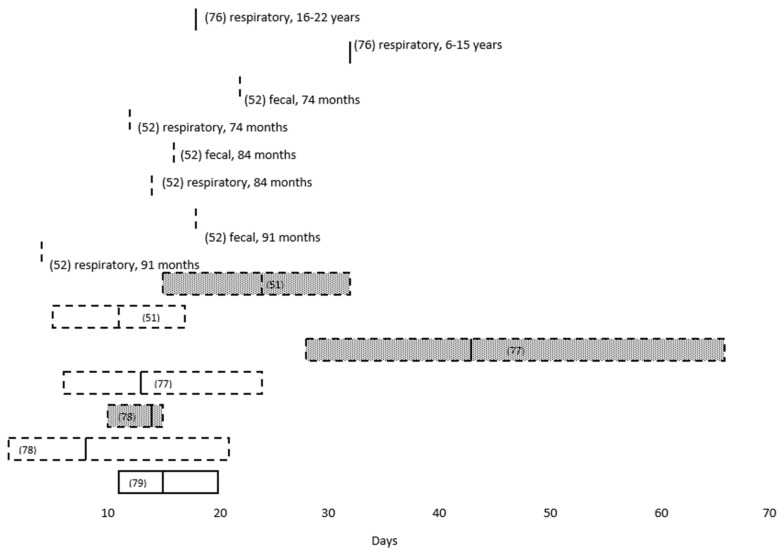
Duration of SARS-CoV-2 RT-PCR positivity in respiratory and fecal specimens reported in studies of pediatric COVID-19 patients cited in this review. The medians are indicated by a solid vertical line, and the means are indicated by a dashed vertical line. A solid box indicates interquartile range, and a dashed box indicates range. Unshaded boxes indicate respiratory specimens, and stippled boxes indicate fecal specimens. The study reference numbers are in parentheses. See text for additional details.

The extent to which children infected with SARS-CoV-2 transmit the virus to others is not yet known. However, a number of studies have demonstrated prolonged clinical shedding by pediatric patients (Table 2), with an inverse correlation between the duration of RT-PCR positivity and age [52,76]. Moreover, detection of virus by RT-PCR tends to be longer in fecal than in respiratory specimens (Figure 4) [51,52,77,78], and at least one study found the duration of clinical shedding to be longer among symptomatic than asymptomatic children [79]. 

Despite these findings, as well as published case reports documenting pediatric transmission of SARS-CoV-2 between children and from children to adults [80], there is limited evidence that children play a prominent role in propagating COVID-19. Several epidemiological studies of households, schools, and daycare settings suggest that children are rarely the index case and that secondary attack rates may be lower when the infector is a child [81]. In one analysis of thirty-one cases of household transmission of SARS-CoV-2 in southeast and southwest Asia, only three (9.7%) identified a child as the index case [82]. In a similar study of thirty-nine Swiss households, only three (8%) familial clusters began with a symptomatic child [83]. However, neither of these studies exclude the possibility of transmission from an asymptomatic child to others in the household.

A limited role for children in the propagation of COVID-19 is also suggested by several school-based studies. In one study of SARS-CoV-2 transmission among children and staff in fifteen schools and ten early childhood education and care settings in the Australian state of New South Wales, there were just eighteen secondary cases identified among 1448 contacts [84]. Similarly, in Sweden, where primary schools and day care centers have remained open during the pandemic, the cumulative incidence for hospitalization with a non-incidental diagnosis of COVID-19 among children in Stockholm younger than 17 years was nine per 100,000, compared to 230/100,000 hospitalized adults during the same time period [85]. Lastly, in one meta-analysis of outbreaks in China, Hong Kong, and Singapore, mathematical modeling suggested that school closures would prevent only 2–4% of COVID-19-related deaths [86], a conclusion also drawn in a study of epidemic data from China, Italy, Japan, Singapore, Canada and South Korea [87]. Nonetheless, as of 18 March 2020, 107 countries had implemented national school closures in response to the pandemic, a number that had fallen to 23 by 25 November 2020 [88]. 

## 9. Persistent Clinical Shedding, Relapse, and Reinfection

The detection of SARS-CoV-2 virus in respiratory specimens from recovered COVID-19 patients after one or more negative RT-PCR assays has been reported [89,90,91,92], raising the question as to whether this represents imperfect sampling, the limited sensitivity of the assay, intermittent shedding, relapse, or reinfection. Although data are limited, there are several small follow up studies of such individuals showing a lack of transmission to family members after the patients were discharged from the hospital, suggesting that they were clinically shedding inert virus [92]. Nonetheless, there are a small number of case reports of recovered COVID-19 patients clinically shedding virus that is genetically distinct from that which was originally isolated [93,94,95,96], a finding that is consistent with either reinfection or mutation of the original virus.

The extent to which detectable virus after a negative assay (i.e., re-positive) represents reinfection has not been clearly established. However, in one study of 87 patients in Guangdong, China, who retested positive, culturable virus or intact genomes consistent with possible reinfection were found in only 14% of cases, and the majority of patients were thought to be clinically shedding inert virus [97]. Moreover, in at least one animal study, nonhuman primates recovering from COVID-19 were protected from reinfection when challenged with the virus [98]. Collectively, these and other studies suggest that in most COVID-19 cases, persistent clinical shedding is not due to reinfection. An alternative explanation is that viruses might be sequestered somewhere in the body (e.g., in extracellular double-membrane vesicles or exosomes) and are released during a second round of cellular shedding [99]. This mechanism has been proposed for certain viruses, including Human Immunodeficiency Virus and Epstein Barr Virus [100]; and although such structures have been observed in cultured SARS-CoV-2-infected cells, their role in spreading the virus remains speculative [99]. Lastly is the possibility of latent virus reactivation, as is seen with the herpes viruses, a phenomenon that was described in a COVID-19 patient who underwent treatment for B cell acute lymphoblastic leukemia [101].

## 10. Discussion

It has been one year since the novel coronavirus provisionally designated 2019-NCoV and subsequently renamed SARS-CoV-2 emerged from Wuhan, China. Since then, the virus has spread globally, and as of 23 November 2020, 59,127,000 COVID-19 cases with 1,396,017 deaths in 215 countries, territories, and conveyances have been reported [15]. COVID-19 has dominated the news and articles abound on transmission and case fatality rates, the utility of masking and social distancing, the efficacy (or lack thereof) of therapeutics, the pursuit of vaccines, as well as the timing of reopening schools and loosening of social restrictions. The virus has similarly become an intense focus of scientific research, with 70,731 results and 41,958 results in PubMed, using the search terms “COVID-19” and “SARS-CoV-2”, respectively (accessed 4 November 2020). We have made significant strides in understanding both the virus and the disease it causes, and several fast-tracked vaccine candidates are currently in Phase 3 clinical trials [102]. However, many fundamental questions have yet to be answered, including the nature and duration of immunity, the long-term sequelae of infection, as well as the transmission risk posed by prolonged clinical shedding by convalescent persons. Regarding the latter, the persistent detection of SARS-CoV-2 in the bodily fluids and feces of convalescent patients introduces a measure of uncertainty with respect to their clinical management. At what point, for example, is it entirely safe to return an elderly recovered COVID-19 patient to a skilled nursing facility, or a cancer patient who has recovered from COVID-19 to the hematology/oncology clinic waiting room? Similarly, when is it appropriate to permit a child with persistently detectable virus in the stool to return to school or day care? Furthermore, what is the significance of a repeat positive SARS-CoV-2 RT-PCR assay on a respiratory sample from a recovered COVID-19 patient in the setting of a prior negative test? These are precisely the types of scenarios confronting physicians and public health officials and for which they are seeking evidence-based guidance.

Certainly, further studies are needed to determine both the mechanism of viral persistence and its implications for safeguarding public health. Nonetheless, based on the current available data, several conclusions can be inferred. Firstly, persistent detection of virus by RT-PCR in respiratory specimens collected from convalescent COVID-19 patients is a common phenomenon, as reported in the studies cited in this paper. In this respect, SARS-CoV-2 is like several other human coronaviruses including SARS-CoV and MERS-CoV [103]. However, among these viruses, MERS-CoV differs from SARS-CoV and SARS-CoV-2 in that prolonged detection of MERS-CoV viral RNA in the stool of infected individuals has not been widely reported [104]. Secondly, the persistent detection of SARS-CoV-2 in clinical specimens is unlikely to reflect either relapse or reinfection in most cases. This statement is supported by a number of studies showing that replication-competent virus is generally not recoverable after ten days following symptom onset in mild to moderate cases of COVID-19 [105] and after twenty days in severe or immunocompromised cases [106]. Similarly, in studies of individuals who have recovered from COVID-19 and subsequently redeveloped symptoms, replication-competent virus was not recoverable, even in the setting of a positive RT-PCR assay [97]. These data, combined with contact tracing studies of people exposed to convalescent COVID-19 patients, prompted the CDC to revise its guidelines regarding the discontinuation of isolation precautions from a test-based to a time-based approach [107].

For several reasons, children infected with SARS-CoV-2 represent a demographic of COVID-19 patients that has garnered much attention. These include observations that although virus remains persistently detectable by RT-PCR in the stool of children with COVID-19 [108], they tend to be less affected than adults, account for a relatively small percentage of diagnosed cases, and are rarely the index case in a household cluster [83]. Nonetheless, the extent to which children propagate COVID-19 is an open question, and with every spike in COVID-19 cases, school districts ponder closures and the implementation of virtual classroom instruction [109]. Although there is no consensus on the best approach, proponents for and opponents of face-to-face instruction agree that there are significant advantages to having children return to the classroom. Some of these have been explicated by The CDC [110] and in a position statement the American Academy of Pediatrics “strongly advocates that all policy considerations for the coming school year should start with a goal of having students physically present in school” [111]. Nonetheless, one can anticipate that pending additional studies of the role of children in transmitting SARS-CoV-2, as well as the release of an effective COVID-19 vaccine, school districts will likely act reflexively to the local prevalence of COVID-19 in their communities.

Although it may seem as if the COVID-19 pandemic is interminable, it is only one year since the virus emerged, and the reality is that Nature does not readily give up her secrets. Hypotheses must be formulated and tested; results must be interpreted and reconciled; and conclusions must withstand the tincture of time. Consider, for example, that it was four years into the AIDS pandemic before the first HIV drug (zidovudine or AZT) was approved, and twelve years until the discovery of potent Highly Active Antiretroviral Therapy (i.e., HAART or “AIDS cocktails”). Similarly, reliably effective drugs against the hepatitis C virus, which was identified in 1989, were only available since 2013 [112]. Certainly, our understanding of SARS-CoV-2 will increase with time, but just assuredly, new questions will also arise. Perhaps there is some consolation to be found in the words of Jules Verne: “Science, my boy, is made up of mistakes, but they are mistakes which it is useful to make, because they lead little by little to the truth [113]”.

## Figures and Tables

**Table 1:** Factors associated with viral persistence in the clinical specimens from COVID-19 patients.

Study (Reference)	Number Studied (N)	Median Duration in Respiratory Specimens (Days)	Independent Factors Associated with Viral Persistence
Zhou F, Yu T, Du R, et al. [32]	191	20.0 days (IQR 17.0–24.0)	NE
Zhou B, She J, Wang Y, Ma X. [33]	41	31 (IQR 24.0–40.0)	NE
Xiao AT, Tong YX, Zhang S. [40]	56	24 (IQR, 18–31)	NE
Zheng S, Fan J, Yu F, et al. [34]	96	18 (Range 13–29)	(1) severe disease (21 days, 14–30 days)
			(2) age > 60 years
			(3) male gender
Fang Z, Zhang Y, Hang C, Ai J, Li S, Zhang W. [35]	32	22.25 ± 3.62 (ICU) vs. 15.67 ± 6.68 (non-ICU)	ICU hospitalization
Li TZ, Cao ZH, Chen Y, et al. [39])	101	11 (IQR 8–14.3)	(1) temperature > 38.5 °C) (OR 5.1, 95%CI: 1.5–18.1)
			(2) corticosteroid use (OR 6.3, 95%CI: 1.5–27.8)
			(3) time from onset to hospitalization (OR 1.8, 95%CI: 1.19–2.7)
Kaijin Xu, Yanfei Chen, Jing Yuan, et al. [41]	113	17 (IQR, 13–22)	(1) male gender (OR, 3.24; 95% CI, 1.31–8.02)
			(2) invasive mechanical ventilation (OR, 9.88; 95% CI, 1.11–88.02)
			(3) time from illness onset to hospital admission (odds ratio [OR], 1.30; 95% confidence interval [CI], 1.10–1.54; *p* = 0.002)

NE: Not examined.

**Table 2:** Clinical Shedding of SARS-CoV-2 by pediatric COVID-19 patients.

Study (Reference)	Number Studied (N)	Median Age	Median Duration in Respiratory Specimens (Days)	Median Duration in Fecal Specimens (Days)	Median Time to Seropositivity (Days)
Bahar B, Jacquot C, Mo YD, et al. [76]	6369		32 (ages 6–15 years)	NE	18 (36 days for “adequate levels” of neutralizing antibodies)
			18 (ages 16–22 years)		
Santos VS, Gurgel RQ, Cuevas LE, et al. [52]	36	74 months (mean)	12 (mean)	22 (mean)	NE
		56 months (mean)	NE	NE	
		84 months (mean)	14.3 (mean)	16.3 (mean)	
		91 (mean)	3.9 (mean)	18.1 (mean)	
Xu CLH, Raval M, Schnall JA, et al. [51]	69		11.1 ± (mean) ± 5.8	23.6 ± (mean) ± 8.8	NE
Liu P, Cai J, Jia R, et al. [77]	9		13 (range 6–24)	43 (range 28–66)	12.9
De Ioris MA, Scarselli A, Ciofi Degli Atti ML, et al. [78]	22	84 months (range 8 days–210 months)	8 (range 1–21)	14 (range 10–15)	NE
Lu Y, Li Y, Deng W, et al. [79]	110	6 years	15 (IQR 11–20)	NE	NE
			11 (asymptomatic)		
			17 (symptomatic)		
